# Microwave-assisted technologies for recycling and regeneration of spent lithium-ion batteries

**DOI:** 10.3389/fchem.2026.1838785

**Published:** 2026-05-07

**Authors:** Jiawei Wu, Shaodong Yang, Sihua Jiang, Hongtao Cui, Jiali Fu, Shijie Gu, Zhan Liu, Min Hong, Yuanlong Liu, Tiefeng Liu

**Affiliations:** 1 Quzhou Institute of Power Battery and Grid Energy Storage, Quzhou, China; 2 Quzhou Institute of Power Battery and Grid Energy Storage, Quzhou, China; 3 Tianjin B&M Science and Technology Co.,Ltd, Tianjin, China; 4 Zhejiang Times Li-ion Material Co.,Ltd, Quzhou, China; 5 College of Chemical and Biological Engineering, Zhejiang University, Hangzhou, China

**Keywords:** carbothermal reduction, electrode regeneration, hydrometallurgical leaching, microwave-assisted recycling, spent lithium-ion batteries

## Abstract

The rapid growth of lithium-ion battery (LIB) deployment in electric vehicles and energy storage systems has led to an increasing volume of end-of-life batteries, highlighting the urgent need for efficient and sustainable recycling technologies. Microwave thermal treatment has recently emerged as a promising approach for LIB recycling due to its unique internal heating mechanism, rapid heating rate, and high energy efficiency. This review systematically summarizes the recent progress of microwave-assisted technologies in the recycling and regeneration of spent LIB materials. The fundamental principles of microwave-matter interaction are first introduced, followed by a comprehensive discussion of its applications in key recycling processes, including pretreatment, carbothermal reduction, and hydrometallurgical leaching. Microwave heating can significantly accelerate the decomposition of binders, enhance reduction reactions, and improve leaching kinetics, thereby enabling faster metal recovery with lower energy consumption. In addition, microwave-assisted strategies for the direct regeneration of degraded electrode materials, such as graphite anodes and cathode materials (e.g., LiCoO_2_ and LiFePO_4_), are highlighted. Overall, microwave technology provides an energy-efficient and low-carbon pathway for the high-value recycling of spent LIBs and represents a promising direction toward establishing sustainable battery recycling systems.

## Graphical Abstract

**Figure:**
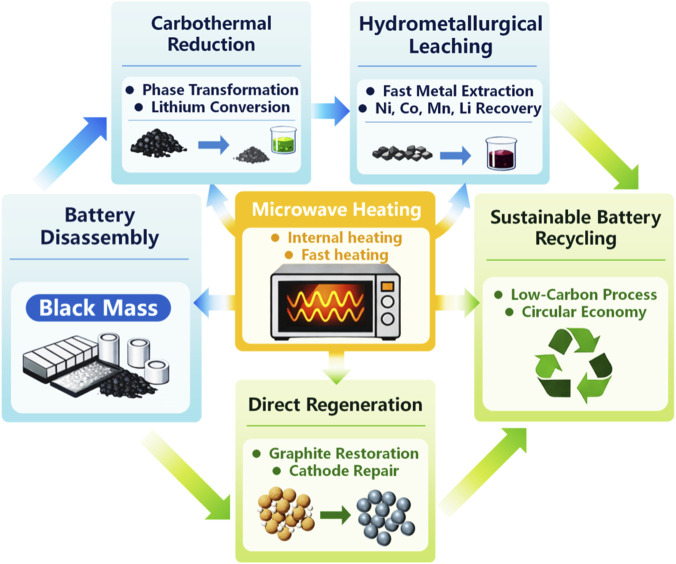
TOC Microwave-assisted technologies enable efficient pretreatment, reduction, leaching, and regeneration of spent lithium-ion batteries, providing a low-carbon pathway for sustainable battery recycling.

## Highlights

- Microwave heating enables rapid and energy-efficient recycling of spent lithium-ion batteries.
- Microwave-assisted processes significantly enhance metal recovery from battery black mass.
- Microwave regeneration restores graphite and cathode materials for high-value recycling.

## 1 Introduction

Lithium-ion batteries (LIBs) have become the dominant technology for energy storage for portable electronics and electric vehicles as well as are making in-roads into grid-scale energy storage because of the well-known advantages of high energy density, long cycle life, and relatively low cost (Zhang J. Y. et al., 2024; Zhang et al., 2025). Notably, the global production capacity of LIBs is expected to reach approximately 8,528 GWh by Venditt and Smith (2025). However, the service life of most LIBs typically ranges between 5 and 8 years, meaning that large volumes of end-of-life LIBs will inevitably emerge in the coming decade (Li Q. W. et al., 2025; Li and Xue, 2025). Rapid accumulation of spent LIBs brings serious environmental challenges and in turn creates strategic resource opportunities (Gu et al., 2017). The former establishes that improper disposal of spent LIBs may lead to the release of toxic electrolytes and heavy metals, posing severe risks to ecosystems and human health (Chen et al., 2025). The latter however presents a good close-loop resource for the battery industry because these batteries contain valuable metals such as lithium, nickel, cobalt, and manganese (Ma et al., 2024). In this end, Governments and international organizations have introduced increasingly strict policies and regulatory frameworks to promote battery recycling and circular supply chains (Wang et al., 2023). For example, recent regulations require companies to account for carbon emissions throughout the entire battery life cycle, including production, use, and recycling stages (European Union, 2026; Hasan et al., 2025; Ngoy et al., 2025). Consequently, developing recycling technologies that minimize both energy consumption and environmental impact has become a key priority for achieving sustainable battery management (Lan et al., 2025).

Currently, recycling strategies for spent LIBs can generally be categorized into three main approaches: pyrometallurgical recycling, hydrometallurgical recycling, and direct regeneration (Wu et al., 2023). Pyrometallurgical processes typically involve high-temperature smelting of battery materials under inert or reducing atmospheres, converting metal oxides into metallic alloys (Reinhart et al., 2023; Woeste et al., 2024). Although this approach is robust and suitable for large-scale industrial operations, it requires extremely high energy input and often results in significant Li and Al losses, which are transferred into slag phases (Cornelio et al., 2024a). Hydrometallurgical processes, in contrast, rely on selective leaching using inorganic acids, organic acids, or deep eutectic solvents to dissolve valuable metals from battery black mass (Shi et al., 2025). Subsequent purification and separation processes-including solvent extraction, precipitation, and crystallization-enable the recovery of high-purity metal salts suitable for the synthesis of new cathode materials (Petzold et al., 2025; Wang J. et al., 2025). Despite its high recovery efficiency, hydrometallurgical recycling consumes large quantities of chemical reagents and may generate secondary waste streams (Wang J. W. et al., 2025). In addition, integrated pyro-hydrometallurgical processes have been proposed to overcome above limitations, a preliminary thermal treatment step-often involving carbothermal reduction-is used to convert layered cathode materials into more leachable phases, followed by acid leaching and metal separation (Liu et al., 2023). In addition to these extraction-based methods, direct regeneration technologies have recently attracted increasing attention (Wang et al., 2024). These approaches aim to restore the crystal structure and electrochemical performance of degraded electrode materials through relithiation and thermal annealing, thereby preserving the intrinsic value of the original materials (Lin et al., 2025; Mengting et al., 2025).

Among above techniques for recycling spent LIBs, thermal processes play a central role in multiple steps, including pretreatment, carbothermal reduction, acid leaching acceleration, and cathode regeneration. Conventional heating methods rely primarily on external heat transfer mechanisms such as electric furnaces, resistive heating, or steam heating (Gebeyehu et al., 2025; Pindar and Dhawan, 2020a). These techniques heat materials from the surface inward through thermal conduction and convection, which often results in slow heating rates and significant temperature gradients within the reaction system. As a consequence, recycling reactions frequently require several hours to complete, leading to high energy consumption and elevated operational costs. Moreover, inefficient heating processes contribute to increased carbon emissions, which contradict the growing demand for low-carbon recycling technologies.In this context, the development of alternative heating technologies capable of overcoming the limitations of conventional thermal methods is of great importance. Microwave heating has emerged as a promising solution for upgrading existing battery recycling processes (Cornelio et al., 2024a). Unlike conventional heating, microwave irradiation transfers electromagnetic energy directly to materials through interactions with molecular dipoles and charge carriers. Polar molecules and ions oscillate rapidly under the alternating electromagnetic field, converting electromagnetic energy directly into heat. This volumetric heating mechanism allows energy to be generated internally within the material rather than being transferred from an external heat source (Bhattacharyya et al., 2025). As a result, microwave heating offers several distinctive advantages, including rapid heating rates, volumetric and uniform heating, high energy efficiency, and the possibility of selective heating of specific components (Bresolin et al., 2025).

In recent years, microwave-assisted technologies have demonstrated remarkable potential in improving both the efficiency and sustainability of LIB recycling (Pindar et al., 2020). Compared with conventional thermal processes, microwave heating can significantly shorten reaction times, enhance reaction kinetics, and reduce energy consumption because materials such as carbon, transition metal oxides, and polar solvents from battery recycling system exhibit strong microwave absorption properties, enabling efficient coupling between the electromagnetic field and the reaction medium (Zhao Y. Z. et al., 2020). The detailed applications involve with pretreatment of black mass, carbothermal reduction, metal leaching enhancement, and direct regeneration of electrode materials (Diaz et al., 2018).Typically, in hydrometallurgical systems, microwave irradiation has been shown to accelerate leaching reactions by enhancing molecular polarization, mass transfer, and surface activation (Aannir et al., 2025). In carbothermal reduction processes, the strong microwave absorption of carbon materials can enable rapid heating and more uniform reduction reactions. Furthermore, microwave techniques have also been successfully applied to the direct regeneration of electrode materials, including the repair of graphite anodes and the structural restoration of cathode materials such as LiCoO_2_ and LiFePO_4_ (He et al., 2022; Zhou et al., 2021). These regeneration approaches represent a promising pathway toward high-value recycling by preserving the intrinsic structure and functionality of electrode materials (Zhu et al., 2025). Despite these promising developments, several challenges remain for the large-scale implementation of microwave-assisted battery recycling technologies (Bhattacharyy et al., 2025). These include the design of scalable microwave reactors, the control of microwave-material interactions in complex multi-phase systems, and the integration of microwave processes into existing industrial recycling frameworks (Chandrasekhar et al., 2026). Addressing these challenges will require interdisciplinary collaboration across materials science, chemical engineering, and process engineering.

**FIGURE 1:**
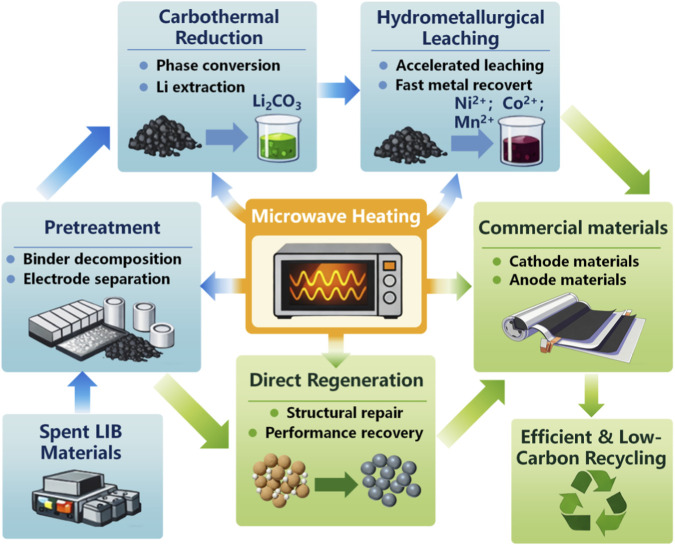
Conceptual framework of microwave-assisted recycling and regeneration of spent LIBs. Microwave heating serves as a central enabling technology throughout the recycling chain, including pretreatment of black mass (binder decomposition and electrode separation), microwave-enhanced carbothermal reduction for phase conversion and lithium extraction, and microwave-assisted hydrometallurgical leaching for efficient metal recovery. In addition, microwave-driven processes enable the direct regeneration of electrode materials, such as graphite anodes and cathode materials. These processes collectively contribute to efficient, low-carbon, and high-value recycling of spent LIBs.

In this review, we systematically summarize the fundamental mechanisms of microwave heating and its emerging applications in the recycling and regeneration of spent LIBs. As illustrated in Figure 1, particular attention is given to the role of microwave technology throughout the entire recycling chain, including pretreatment of battery materials, microwave-enhanced carbothermal reduction, microwave-assisted hydrometallurgical leaching, and direct regeneration of electrode materials. In addition, the advantages of microwave processing over conventional heating methods are analyzed in terms of reaction kinetics, energy efficiency, and environmental impact. Finally, future perspectives and research directions for the industrial implementation of microwave-assisted recycling technologies are discussed, highlighting their potential to enable low-carbon recycling and high-value material recovery for a sustainable battery economy.

## 2 Fundamentals of microwave heating

**FIGURE 2:**
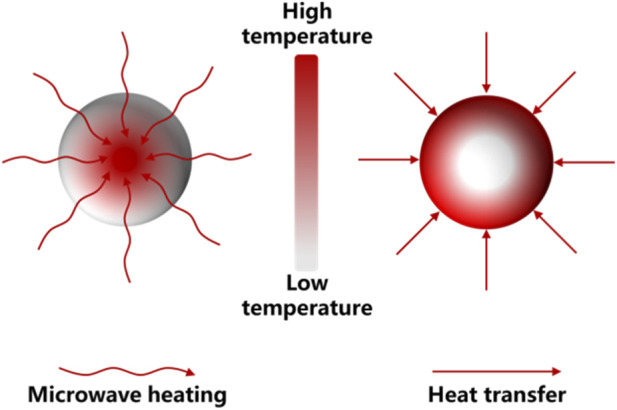
Schematic comparison between conventional thermal conduction heating and microwave heating mechanisms.

Microwave heating is an advanced thermal processing technique that has attracted significant attention in materials processing and chemical engineering due to its unique heating mechanism and high energy efficiency. Unlike conventional heating methods, which rely on heat transfer from external heat sources to materials through conduction, convection, and radiation, microwave heating generates heat directly within materials through electromagnetic interactions (Sun et al., 2016). As shown in Figure 2, this volumetric heating mechanism allows microwave energy to be converted directly into thermal energy inside the reaction system, resulting in rapid heating rates, improved energy utilization efficiency, and more uniform temperature distribution. Microwaves are electromagnetic waves with frequencies ranging from 300 MHz to 300 GHz. In most industrial and laboratory microwave heating systems, a frequency of 2.45 GHz is commonly used to avoid interference with communication signals. When microwave radiation interacts with materials, energy is absorbed by dielectric materials and converted into heat through several mechanisms, primarily dipole polarization and ionic conduction (Kuang et al., 2019). Dipole polarization occurs when polar molecules attempt to align with the alternating electromagnetic field of the microwave radiation. Because the electric field oscillates rapidly, the polar molecules continuously reorient themselves to follow the field direction. This repeated reorientation leads to molecular friction and energy dissipation, which is converted into heat. Materials containing polar molecules, such as water, organic solvents, and certain electrolytes, therefore exhibit strong microwave absorption capability (Bresolin et al., 2025; Zhang G. H. et al., 2024). In addition to dipole polarization, ionic conduction also contributes significantly to microwave heating in systems containing mobile charge carriers. Under the influence of the oscillating electromagnetic field, ions migrate back and forth, generating resistive heating through collisions with surrounding molecules (Fulo et al., 2020). This mechanism is particularly relevant in chemical reaction systems and battery materials that contain conductive species or ionic compounds (Sun et al., 2016).

The efficiency of microwave heating is largely determined by the dielectric properties of materials, including the dielectric constant and dielectric loss factor. The dielectric constant reflects the ability of a material to store electromagnetic energy, whereas the dielectric loss factor describes the ability of the material to convert electromagnetic energy into heat (Lv et al., 2022). Materials with high dielectric loss factors generally exhibit stronger microwave absorption and higher heating efficiency (Kim et al., 2014). In LIB recycling systems, components such as carbon materials, transition metal oxides, and certain solvents possess favorable dielectric properties, enabling efficient coupling with microwave radiation (Sharma et al., 2024). Another vital parameter in microwave heating is the penetration depth, which describes how deeply microwave energy can penetrate into a material before being attenuated. Penetration depth depends on the frequency of the microwave radiation and the dielectric properties of the material (Salakhi and Thomson, 2025). Materials with excessively high dielectric losses may absorb microwave energy too strongly at the surface, resulting in shallow penetration depth and non-uniform heating. Therefore, optimizing material composition and reactor design is essential to ensure effective microwave energy distribution during processing. As shown in Table 1, compared with conventional heating technologies, microwave heating offers several distinctive advantages (Wang Z. X. et al., 2025). First, microwave irradiation provides rapid heating, allowing reaction temperatures to be reached within minutes rather than hours. Second, the volumetric heating mechanism enables more uniform temperature distribution, reducing thermal gradients within the reaction system. Third, microwave heating can exhibit selective heating behavior, where microwave-absorbing components are preferentially heated, potentially enhancing reaction kinetics and process efficiency.

**TABLE 1:** Comparison between microwave heating and conventional heating in LIB recycling processes.

Heating method	Heating mechanism	Energy transfer mode	Heating rate	Temperature uniformity	Reaction time	Energy efficiency	Typical applications in LIB recycling
Conventional heating (furnace/resistive)	External heat conduction/convection/radiation	Surface-to-core	Slow	Non-uniform (temperature gradients)	Typically hours	Relatively low (heat loss)	Pyrometallurgical smelting, thermal pretreatment, calcination
Microwave heating	Electromagnetic waves coupling with polar/conductive materials	Volumetric internal heating	Rapid	More uniform (throughout materials)	Minutes to tens of minutes	High (direct energy conversion)	Black mass pretreatment, carbothermal reduction, microwave-assisted leaching, electrode regeneration
Hybrid microwave–conventional heating	Microwave + external heating	Internal + external coupling	Moderate–rapid	Improved	Tens of minutes	Higher than conventional heating	Advanced carbothermal reduction and large-scale microwave reactors

In addition to the well-known rapid heating effect (thermal effect), microwave irradiation can also directly influence chemical reaction pathways and mass transfer behavior through non-thermal effects. Studies have revealed that microwave electromagnetic fields can be stored directly within reactant molecules, enhancing their internal energy and thereby lowering reaction energy barriers to promote reactions. Furthermore, microwave electric fields can induce dipole polarization of water molecules, disrupting the stable tetrahedral hydrogen-bond network and transforming it into a chain-like structure. Therefore, microwave irradiation may also enhance the interaction between solvents and metal ions by altering the hydrogen-bond network structure of polar solvents (such as water, organic acids, and deep eutectic solvents), thereby improving leaching kinetics and selectivity.

Relying on these advantages, microwave heating has been increasingly applied in various stages of LIB, recycling processes, including pretreatment of battery materials, carbothermal reduction, hydrometallurgical leaching, and electrode regeneration. Understanding the fundamental principles governing microwave–matter interactions is therefore essential for optimizing microwave-assisted recycling technologies and improving process efficiency.

## 3 Applications of microwave thermal treatment in metal recovery and resource utilization of battery black mass

Within the dominant pyro-hydrometallurgical recycling framework, microwave thermal treatment has emerged as a promising strategy for enhancing reaction efficiency and improving process selectivity. Owing to its intrinsic volumetric heating characteristics, microwave irradiation enables rapid and uniform heating throughout the reaction system, thereby accelerating chemical reactions and improving energy utilization efficiency (Biswal et al., 2024).

In the recycling of LIBs, microwave technology can be integrated into multiple stages of the resource recovery process. These include pretreatment of battery materials, carbothermal reduction for phase transformation, and microwave-assisted hydrometallurgical leaching for metal extraction (Ko et al., 2024; Zhou et al., 2025). Compared with conventional external heating methods, microwave heating can significantly shorten reaction times, enhance mass transfer, and improve the overall efficiency of metal recovery processes (Zanoletti et al., 2024). Conventional thermal processes typically rely on heat conduction from external heat sources, which often leads to slow heating rates, temperature gradients within materials, and inefficient energy utilization (Fu et al., 2020; Pindar and Dhawan, 2020b). These limitations can hinder reaction kinetics and increase energy consumption. Microwave-assisted processing provides an effective alternative by delivering energy directly into materials through microwave-matter interactions. Therefore, microwave thermal treatment offers new opportunities for improving the efficiency and sustainability of LIB recycling technologies. This section summarizes recent advances in microwave-assisted resource recovery from battery black mass, focusing on three key stages: pretreatment, carbothermal reduction, and hydrometallurgical leaching.

### 3.1 Microwave-assisted pretreatment of battery black mass

The primary objective of the pretreatment stage is to separate electrode materials from current collectors and remove organic components such as electrolytes and polymer binders (Liu et al., 2023). In spent LIBs, cathode active materials are typically tightly attached to aluminum foil through polyvinylidene fluoride (PVDF) binders, which exhibit high chemical and thermal stability (Woeste et al., 2024). This strong adhesion makes the separation of active materials from current collectors challenging (Wang et al., 2025a). Conventional pretreatment methods commonly involve low-temperature thermal decomposition, which typically requires heating for several hours to degrade PVDF binders. However, this process not only consumes significant energy but also may lead to oxidation of metallic components such as copper and aluminum, resulting in impurities that reduce the purity of the recovered black mass (Jiang et al., 2023). In addition, the decomposition of organic components during thermal treatment may release hazardous gases such as HF, CO, and volatile electrolyte solvents, posing environmental and safety concerns (Diaz et al., 2018).

**FIGURE 3:**
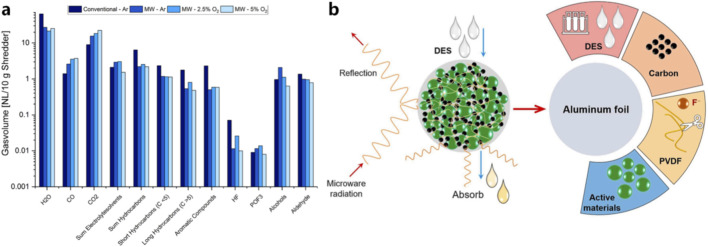
Microwave-assisted processes in battery recycling: **(a)** gas evolution during different thermal treatment processes (Stallmeister et al., 2025); **(b)** reaction mechanism of PVDF degradation under microwave heating (Li P. et al., 2024).

Microwave heating provides an efficient alternative for accelerating binder decomposition and material separation (Xu et al., 2021). Due to the strong microwave absorption properties of carbon materials and polar compounds in battery black mass, microwave irradiation can rapidly heat the material from within, significantly accelerating the decomposition of organic components. For example, As shown in Figure 3a, microwave-assisted pyrolysis has been reported to improve the decomposition efficiency of PVDF binders while reducing the generation of harmful gaseous byproducts (Stallmeister et al., 2025).

Furthermore, microwave heating can enhance the separation efficiency of cathode materials from aluminum foil (Li P. et al., 2024). Li et al. employed a deep eutectic solvent combined with microwave heating, achieving efficient and complete separation of aluminum foil from cathode materials within just 8 min at 120 °C (Figure 3b) Because cathode active materials exhibit relatively high dielectric properties and absorb microwave energy effectively, while aluminum foils tend to reflect microwave radiation, selective heating can be achieved, enabling efficient detachment of active materials without excessive oxidation of aluminum. These advantages make microwave-assisted pretreatment a promising strategy for improving the efficiency and environmental performance of battery recycling processes (Siame et al., 2025).

### 3.2 Microwave-assisted carbothermal reduction

**FIGURE 4:**
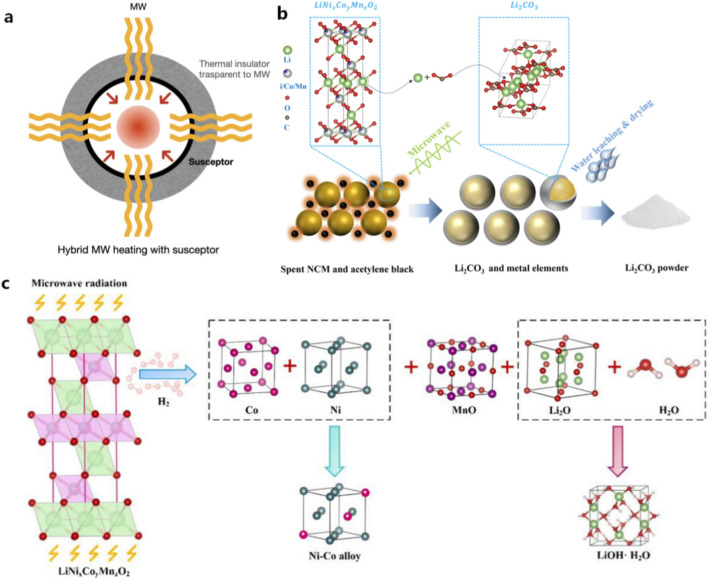
Microwave-assisted processes in battery recycling: **(a)** schematic illustration of a microwave reaction chamber (Fahimi et al., 2023); **(b)** microwave-assisted carbothermal reduction and subsequent water leaching process (Tong et al., 2025); **(c)** phase transformation of spent cathode materials during microwave-assisted hydrogen reduction (You et al., 2023).

Carbothermal reduction is an important step in many LIB recycling processes, particularly in combined pyro-hydrometallurgical recycling routes. During this stage, battery black mass is thermally treated in the presence of carbonaceous materials, which act as reducing agents to convert metal oxides into more easily extractable phases. Generally, high temperatures (often above 900 °C) are required to achieve effective reduction reactions, in order to enable the heat diffusion from the external environment to the interior of the material through conduction. However, as reduction proceeds, reaction products may form a dense layer around the particle surface, hindering further reaction and resulting in incomplete phase transformation or prolonged reaction times. By contrast, microwave heating offers several advantages for carbothermal reduction processes. Carbon materials present in battery black mass exhibit excellent microwave absorption properties and can act as efficient microwave susceptors. As a result, microwave irradiation can directly heat carbon particles and surrounding materials, leading to rapid and uniform temperature increases throughout the reaction system (Figure 4a) (Fahimi et al., 2023). Furthermore, microwave-assisted carbothermal reduction also provides an efficient pathway for converting cathode materials into more leachable phases while reducing energy consumption compared with conventional thermal reduction processes (Zhang et al., 2026).

This volumetric heating mechanism enhances reduction kinetics and improves reaction efficiency. Several studies have demonstrated that microwave-assisted carbothermal reduction can significantly shorten reaction time while achieving high lithium conversion efficiency. As shown in Figures 4b,c, microwave irradiation can convert lithium into lithium carbonate or lithium oxide, allowing for separate extraction. Combined with water leaching, it can achieve nearly 90% lithium recovery in a few minutes of processing time (Tong et al., 2025; You et al., 2023). In addition, the type, source, and amount of carbon material have a decisive impact on the reduction effect. Graphite is an excellent microwave absorber and reducing agent, enabling efficient heating without external addition, but a relatively high dosage (approximately 30%) is required to ensure complete reduction (Pindar and Dhawan, 2020c). Acetylene black, due to its high electrical conductivity, can efficiently convert electromagnetic energy into thermal energy, achieving high selective lithium leaching with a lower dosage (15%–25%), offering the highest efficiency (Zhang et al., 2026). Biomass-derived carbon (e.g., macadamia nut shells) is green and sustainable, and the biochar formed from its pyrolysis can reduce the decomposition temperature of NCM to 300 °C (Zhao Y. et al., 2020). Overall, carbon materials determine the heating rate and temperature distribution under the microwave field through their dielectric loss properties, and control the product phases through their reducing ability, thereby affecting subsequent separation efficiency. Optimizing the type and dosage of carbon materials is key to achieving low-temperature, rapid, and highly selective metal recovery.

### 3.3 Microwave-assisted hydrometallurgical leaching

**FIGURE 5:**
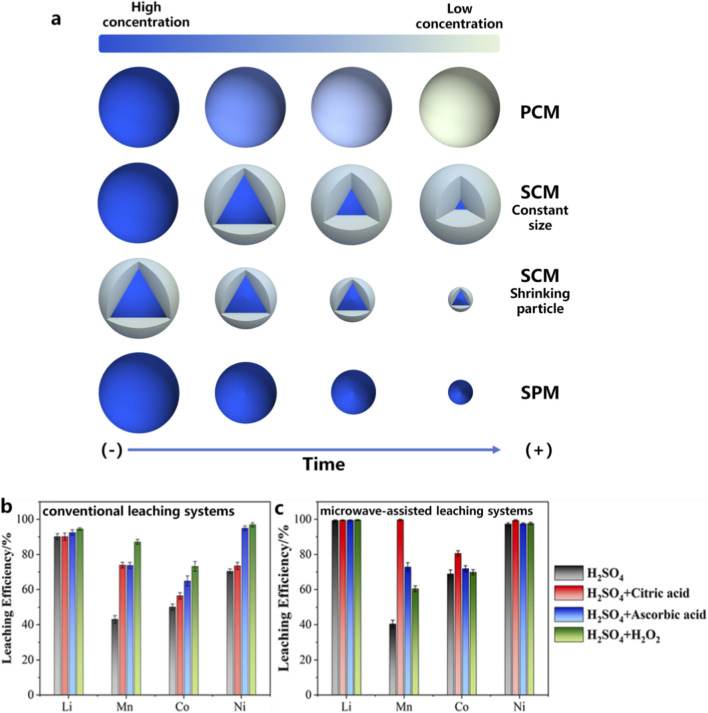
**(a)** Typical kinetic models describing metal leaching processes in hydrometallurgical recycling of LIBs; Microwave-assisted leaching processes for battery recycling: **(b,c)** comparison between conventional and microwave-assisted leaching systems (Li S. et al., 2024).

Hydrometallurgical leaching is currently the most widely used method for recovering valuable metals from spent LIBs (Jain et al., 2024). In this process, valuable metal elements contained in battery black mass are dissolved into solution through chemical reactions with leaching agents, followed by purification and separation processes (Billy et al., 2018). The leaching step is particularly critical because it directly determines the recovery efficiency of valuable metals and significantly influences the overall economic and environmental impact of the recycling process (Nasser and Petranikova, 2021). As shown in Figure 5a, Several kinetic models have been proposed to describe the leaching process, including the shrinking core model (SCM), the progressive conversion model (PCM), and the shrinking particle model (SPM) (Faraji et al., 2022).

In many cases, the leaching kinetics of cathode materials are controlled by diffusion through the residual product layer formed during dissolution (Cerrillo-Gonzalez et al., 2020; Liu et al., 2019). Currently, microwave-assisted leaching has been widely investigated as a method to enhance leaching kinetics. Microwave irradiation can directly heat polar solvents and reaction media, resulting in rapid temperature increases and improved mass transfer. In addition, microwave-induced thermal stress can create microcracks and surface etching on particle surfaces, increasing surface area and facilitating contact between solid particles and leaching agents (Li S. et al., 2024). Li used a sulfuric acid-based system with citric acid as a reductant, combined with microwave-assisted leaching (Figures 5b,c), and the microwave-assisted leaching significantly enhanced the leaching efficiencies of Ni, Co, and Mn from 73.5%, 56.4%, and 73.9%–99.4%, 80.6%, and 99.7%, respectively.

**FIGURE 6:**
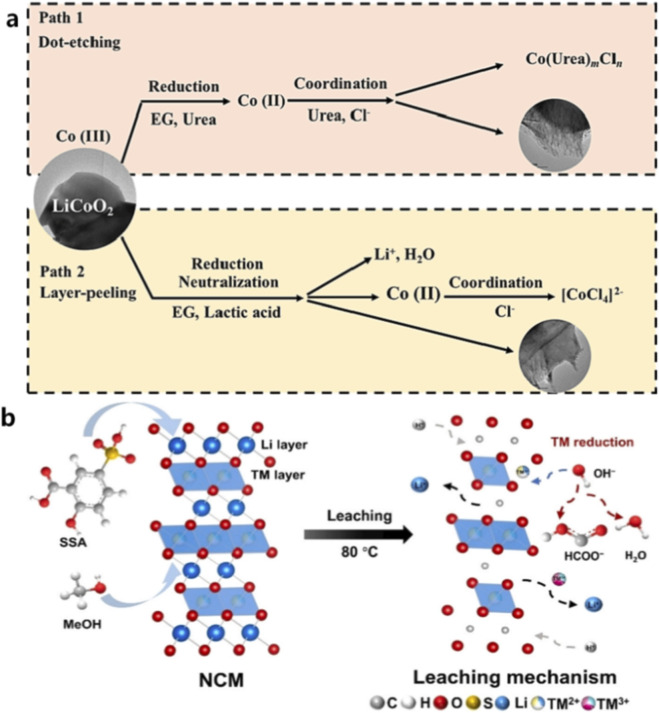
Microwave-assisted leaching processes for battery recycling: **(a)** leaching mechanisms of LiCoO_2_ in different DES systems, including pitting corrosion and layer exfoliation pathways (Zhu et al., 2023); **(b)** mechanism of the methanol-SSA leaching system for NCM cathodes (Hu et al., 2025).

Compared with conventional heating methods, microwave-assisted leaching can significantly shorten reaction times and reduce energy consumption (Alhashim et al., 2024; Dos Santos et al., 2022; Fu et al., 2020; Guimarães et al., 2022; Pindar and Dhawan, 2021). In some reported systems, microwave-assisted leaching has achieved metal recovery efficiencies exceeding 99% within minutes of treatment (Li S. et al., 2024). Moreover, microwave heating can improve the utilization efficiency of chemical reagents by minimizing localized overheating, which may otherwise cause decomposition of additives such as hydrogen peroxide. In addition, microwave-assisted leaching has also been successfully applied to various leaching systems, including inorganic acid systems, organic acid systems, and deep eutectic solvent (DES) systems (Aannir et al., 2025; Hu et al., 2025; Raj et al., 2023; Zhu et al., 2023). As show in Figure 6a, with microwave assistance, Zhu et al. found that under the influence of a microwave electric field, the dipole moments induced by various components of the deep eutectic solvent (DES), such as Ch^+^, ethylene glycol (E.G.,), lactic acid, and urea, on the surface of LiCoO_2_ increased dramatically with the electric field strength. When the electric field intensity reached 2 a.u., the dipole moments of these components were enhanced by a factor of 27–168 compared to those in the absence of an electric field. This enhancement enables DES molecules to exhibit stronger polarization and orientation capabilities under the microwave field, thereby significantly improving their interaction activity with the electrode material surface. Hu et al. adopted a methanol-SSA leaching system combined with microwave heating, and achieved leaching efficiencies above 99% within 15 min at 80 °C (Figure 6b). These results demonstrate that microwave irradiation can significantly enhance reaction kinetics and improve metal recovery efficiency, highlighting its potential for developing more sustainable hydrometallurgical recycling technologies.

### 3.4 Process intensification enabled by microwave heating

Process intensification refers to the development of technologies that dramatically enhance chemical reaction efficiency, reduce energy consumption, and simplify process flows compared with conventional methods. Due to the unique microwave-matter interaction mechanisms, microwave heating can fundamentally alter reaction environments and improve mass and heat transfer processes. One of the key advantages of microwave heating lies in its volumetric heating capability, where electromagnetic energy is directly absorbed by materials and converted into thermal energy within the reaction medium. This mechanism eliminates the dependence on external heat transfer, which often limits reaction rates in conventional thermal systems. As a result, microwave heating can achieve significantly faster temperature ramping and shorter reaction times. Another important feature of microwave-assisted processes is the possibility of selective heating. In heterogeneous reaction systems such as battery black mass, different components exhibit different dielectric properties. Carbon materials and certain transition metal oxides typically possess strong microwave absorption capability, enabling them to be preferentially heated under microwave irradiation. This selective heating behavior can enhance reaction kinetics in targeted phases while minimizing unnecessary heating of surrounding materials. Microwave irradiation can also enhance mass transfer and reaction kinetics. Rapid heating and localized thermal gradients may induce thermal stresses within solid particles, resulting in the formation of microcracks and increased surface area. These structural modifications improve contact between reactants and facilitate diffusion of chemical species during leaching and reduction processes. In addition, microwave irradiation can promote polarization and activation of polar molecules in liquid systems, further accelerating chemical reactions. From an energy perspective, microwave-assisted processes often exhibit higher energy efficiency compared with conventional heating systems. Because energy is delivered directly to the reaction medium rather than heating the entire reactor environment, thermal losses can be significantly reduced. Several studies have reported substantial reductions in energy consumption and reaction time for microwave-assisted recycling processes. Overall, microwave-assisted processing provides an effective pathway for intensifying LIB recycling processes. By improving reaction kinetics, enhancing mass transfer, and reducing energy consumption, microwave technology can significantly increase the efficiency and sustainability of metal recovery and material regeneration processes. These advantages highlight the strong potential of microwave-assisted recycling technologies for next-generation battery recycling systems.

## 4 Microwave-assisted regeneration of degraded LIB materials

In addition to conventional metal recovery routes, the direct regeneration of degraded electrode materials has emerged as a promising strategy for improving the sustainability and economic efficiency of LIB recycling. Traditional recycling processes typically decompose battery materials into elemental metals or salts, which must then be re-synthesized into new electrode materials. Although effective, this approach involves multiple energy-intensive steps and significant chemical consumption. Direct regeneration strategies aim to restore the crystal structure and electrochemical functionality of degraded electrode materials while preserving their intrinsic material value. By repairing structural defects and replenishing lost lithium, the regenerated materials can regain electrochemical performance comparable to that of newly synthesized materials. Among the various regeneration approaches, microwave-assisted technologies have attracted increasing attention due to their rapid heating capability, high energy efficiency, and potential for selective heating. Microwave irradiation enables rapid volumetric heating within electrode materials, which can accelerate defect healing, phase reconstruction, and lithium diffusion processes. Compared with conventional furnace heating, microwave-assisted regeneration often requires significantly shorter processing times and lower energy consumption. Therefore, microwave-driven regeneration has become an emerging approach for the high-value recycling of spent LIB materials.

### 4.1 Microwave-assisted regeneration of graphite anodes

**FIGURE 7:**
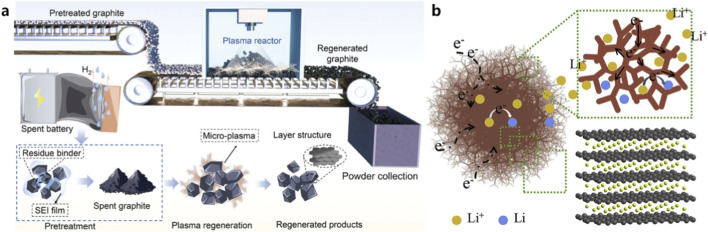
Microwave-assisted regeneration of spent graphite anodes: **(a)** schematic of microwave-induced graphite regeneration (Shan et al., 2024); **(b)** regeneration mechanism (Ma et al., 2018).

Graphite is the most widely used anode material in commercial LIBs. During battery operation, graphite undergoes repeated lithium intercalation and deintercalation processes, which may lead to structural defects, SEI accumulation, and contamination by residual electrolytes and metal impurities (Zheng et al., 2023). As a result, spent graphite anodes often exhibit reduced electrical conductivity and electrochemical performance. Conventional regeneration methods for spent graphite mainly include acid purification and high-temperature graphitization treatments (Xie et al., 2023). Acid treatment can remove metal impurities and residual electrolytes; however, strong oxidizing conditions may damage the graphite structure and introduce additional defects (Xie et al., 2024). High-temperature graphitization processes typically require temperatures exceeding 2500 °C to restore graphite crystallinity, resulting in extremely high energy consumption (Yi et al., 2021). Unlike above conventional methods, microwave-assisted regeneration offers an alternative approach with significantly improved energy efficiency (Lee et al., 2022; Ma et al., 2018). Due to the excellent microwave absorption capability of carbon materials, graphite can be rapidly heated under microwave irradiation, enabling efficient defect repair and impurity removal within a short processing time (Lee et al., 2022; Ma et al., 2018). Microwave heating can induce localized high temperatures and even plasma effects, which facilitate the removal of surface contaminants and promote structural reconstruction of graphite. Several studies have demonstrated that rapid microwave processing can effectively remove surface SEI layers, repair lattice defects, and restore the ordered graphite structure. As a result, microwave-treated graphite can recover electrochemical performance comparable to that of commercial graphite. For example (Figure 7a), Shan et al. processed exfoliated graphite for 30 s under nitrogen atmosphere to achieve efficient regeneration. The regenerated graphite delivered 352.2 mAh·g^-1^ at 0.2 C, with an initial coulombic efficiency of 87% and 81% capacity retention after 400 cycles. Ma et al. reconstructed the surface of waste graphite via microwave exfoliation and spray drying, and the resulting regenerated graphite anode delivered a capacity of 409.7 mAh·g^-1^ after 100 cycles at 0.1 C, with the initial Coulombic efficiency increased from 74.2% to 81.6% (Figure 7b).

### 4.2 Microwave-assisted regeneration of cathode materials

**FIGURE 8:**
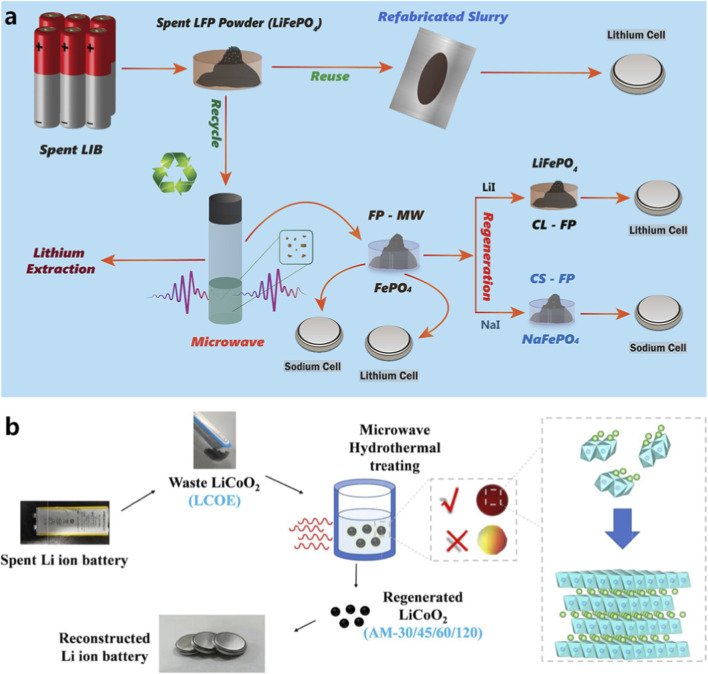
Microwave-driven regeneration of battery materials: **(a)** regeneration of LiFePO_4_ cathode materials (Gangaja et al., 2021); **(b)** microwave-assisted restoration of LiCoO_2_ cathodes (Liu et al., 2022).

Among various recycling strategies, direct regeneration of cathode materials has attracted increasing attention due to its ability to preserve the intrinsic crystal structure of active materials (Garole et al., 2020; Harper et al., 2019; Leal et al., 2023; Li et al., 2018; Refly et al., 2020). Compared with metal extraction processes, direct regeneration can significantly reduce energy consumption and material loss during recycling (He et al., 2022; Wang et al., 2026). Microwave-assisted regeneration has been successfully applied to several cathode materials, including LiCoO_2_ (LCO), LiFePO_4_ (LFP), and ternary cathode materials, as illustrated in the process flow in Figure 8. Microwave heating can promote rapid lithium diffusion and structural reconstruction within cathode particles, enabling efficient relithiation and restoration of the layered crystal structure (Lu et al., 2022; Meng et al., 2019; Wang J. et al., 2022; Wang J. X. et al., 2022). For example, microwave-assisted hydrothermal or solid-state relithiation methods have been used to restore lithium-deficient cathode materials (Gangaja et al., 2021; Jang et al., 2025; Liu et al., 2022). The rapid heating capability of microwave irradiation accelerates lithium diffusion into the cathode lattice, facilitating the reconstruction of the original layered structure. In addition, microwave treatment can repair surface defects and reduce the formation of inactive phases.

**FIGURE 9:**
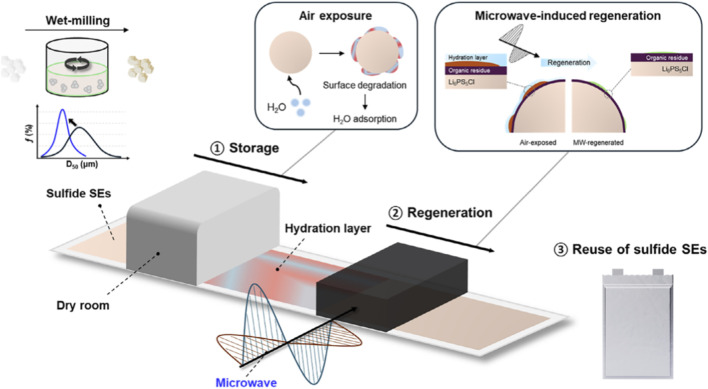
Microwave-assisted recovery of sulfide solid electrolytes (Jang et al., 2025).

Microwave regeneration has also been applied to restore degraded solid-state electrolyte materials. For instance, microwave-assisted treatment has been reported to recover the ionic conductivity of sulfide-based solid electrolytes after exposure to air (Figure 9) (Jang et al., 2025). These studies demonstrate that microwave-assisted regeneration is not limited to electrode materials but may also be extended to other battery components. Overall, microwave-assisted regeneration technologies provide a promising pathway for restoring electrochemical performance while minimizing energy consumption and processing time.

### 4.3 Summary of microwave-assisted material regeneration

**FIGURE 10:**
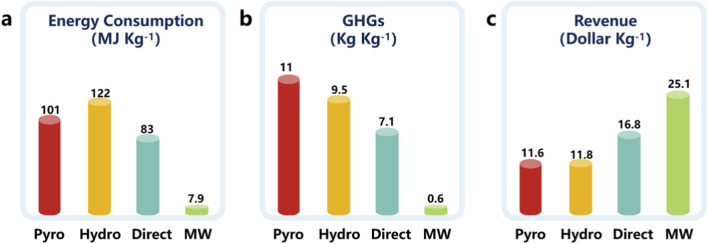
Environmental and economic comparison of different LIB recycling routes based on life-cycle analysis: **(a–c)** comparison of energy consumption, greenhouse gas emissions, and economic profitability.

Currently, microwave-assisted regeneration has emerged as an efficient strategy for the high-value recycling of spent LIB materials. By utilizing the rapid volumetric heating capability of microwave irradiation, structural defects in degraded electrode materials can be effectively repaired, while Li replenishment and phase reconstruction processes can be significantly accelerated. For graphite anodes, microwave-assisted treatment enables efficient removal of SEI layers and restoration of graphite crystallinity, leading to improved electrochemical performance. For cathode materials, microwave-driven relithiation and structural reconstruction can effectively restore lithium storage capability and cycling stability. As shown in Figure 10 and Table 2, compared with conventional high-temperature regeneration processes, microwave-assisted technologies offer substantial advantages in terms of energy efficiency, reaction speed, and environmental sustainability (Hu et al., 2024). Although microwave regeneration technologies show great promise, challenges related to reactor design, process scalability, and uniform heating still need to be addressed before large-scale industrial implementation can be realized. Nevertheless, continued advances in microwave reactor engineering and process optimization are expected to further enhance the feasibility of microwave-assisted regeneration as a key technology for sustainable LIB recycling.

**TABLE 2:** Summary of microwave-assisted processes for recycling and regeneration of spent LIB materials.

Process	Material	Microwave conditions	Recovery/Performance	Key outcome
Carbothermal reduction (Tong et al., 2025)	NCM	500 W, 10 min; water leaching	Li recovery: 88.24%	Energy consumption: 2.6 MWh t^-1^
Carbothermal reduction (Pindar and Dhawan, 2020c)	NCM	900 W, 8 min + citric acid leaching (80 °C, 30 min)	Ni 98%, Co 90%, Mn 98%, Li 94%	Enhanced reduction–leaching efficiency
Carbothermal reduction (Zhao et al., 2020b)	NCM	500 °C–750 °C thermal reduction	Li recovery: 93.4%	Phase conversion improves leachability
Leaching (Li et al., 2024b)	NCM	120 °C, 20 min; H_2_SO_4_ + citric acid	Ni 99.3%, Co 99.5%, Mn 99.7%, Li 99.5%	High-efficiency microwave-assisted leaching
Leaching (Aannir et al., 2025)	NCM:LMO (1:1)	75 °C, 10 min; HNO_3_ + H_2_O_2_	Ni 98.78%, Co 99.46%, Mn 99.63%, Li 98.78%	Rapid leaching kinetics
Leaching (Hu et al., 2025)	NCM	80 °C, 15 min; MeOH–SSA system	Ni 99.68%, Co 99.35%, Mn 99.81%, Li 99.97%	Closed-loop leaching process
Leaching (Hu et al., 2025)	Mixed cathodes	80 °C, 30 min; MeOH–SSA system	Ni 99.99%, Co 99.94%, Mn 99.96%, Li 99.98%, Fe 99.94%	Efficient multi-metal recovery
Leaching (Zhu et al., 2023)	LCO	160 W, 4 min; DES system	Li and Co: ∼100%	Ultrahigh leaching efficiency
Leaching (Zhu et al., 2023)	NCM	220 °C, 30 min; DES system	Li recovery: 99%	Selective Li extraction
Leaching (Cornelio et al., 2024b)	NCM	1000 W, 10 min; malic acid + H_2_O_2_	Ni 72%, Co 82.1%, Mn 87.1%, Li 90.9%	Organic acid-assisted leaching
Regeneration (Shan et al., 2024)	Spent graphite	2.45 GHz, 30 s	352.2 mAh·g^-1^; 81% capacity retention (400 cycles)	Rapid graphite regeneration
Regeneration (Fan et al., 2022)	Spent graphite	900 °C, 60 min	354.1 mAh·g^-1^; 98.3% capacity retention	Structural restoration
Regeneration (Jiang et al., 2022)	Spent graphite	150 °C, 60 min	164.1 mAh·g^-1^; 94.9% retention (100 cycles)	Graphene-assisted regeneration
Regeneration (Li et al., 2025b)	Spent graphite	2.45 GHz, 5 s	188 mAh·g^-1^; 95.2% retention (1000 cycles)	Ultrafast microwave reduction
Regeneration (Gangaja et al., 2021)	LiFePO_4_	250 W, 15 min	145 mAh·g^-1^; 90% retention (200 cycles)	Cathode regeneration
Regeneration (Hu et al., 2024)	LiCoO_2_	600 W, 100 s	140.8 mAh·g^-1^; 88% retention (300 cycles)	Microwave relithiation
Regeneration (Liu et al., 2022)	LiCoO_2_	225 °C, 45 min	141.7 mAh·g^-1^; 94.5% retention (100 cycles)	Hydrothermal–microwave repair
Regeneration (Jang et al., 2025)	LPSCl electrolyte	800 W, 10 min	98.3% ionic conductivity restored	SSE regeneration

## 5 Summary and outlook

**FIGURE 11:**
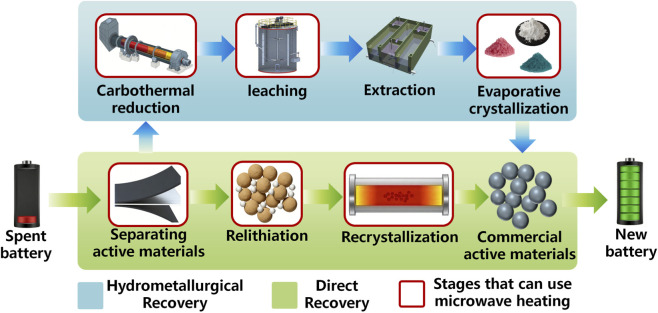
Key stages in the recycling of spent LIBs where microwave heating can be applied, including pretreatment, carbothermal reduction, hydrometallurgical leaching, and electrode regeneration.

In this review, the recent progress in microwave-assisted recycling and regeneration of spent LIBs has been systematically summarized. Compared with conventional heating methods, microwave irradiation offers significant advantages due to its intrinsic volumetric heating mechanism, which enables rapid energy transfer, enhanced reaction kinetics, and improved process efficiency. As illustrate in Figure 11, microwave technology demonstrates broad applicability across the entire battery recycling chain. In pretreatment processes, microwave heating facilitates rapid decomposition of organic binders and efficient separation of electrode materials, thereby improving the purity of black mass. In carbothermal reduction processes, the strong microwave absorption capability of carbon materials enables efficient phase conversion and lithium extraction with reduced energy consumption. In hydrometallurgical recycling, microwave-assisted leaching significantly accelerates reaction kinetics and improves metal recovery efficiency while shortening processing time. Furthermore, microwave-driven regeneration strategies provide promising opportunities for the direct repair of degraded electrode materials, allowing the preservation of material value and reducing overall recycling costs. Overall, microwave-assisted technologies provide a promising pathway toward low-carbon and high-efficiency recycling of LIBs. Continued advances in reactor engineering, mechanistic understanding, and process integration are expected to further promote the industrial implementation of microwave-based recycling technologies and contribute to the development of a sustainable circular battery economy.

Despite the promising advances in microwave-assisted recycling technologies for spent LIBs, several scientific and technological challenges remain before large-scale industrial implementation can be achieved. Addressing these challenges requires interdisciplinary efforts spanning materials science, chemical engineering, and process design. Future research should focus on the following key directions.

### 5.1 Reactor design and scale-up of microwave systems

One of the primary challenges for industrial application lies in the scale-up of microwave reactors. Most current studies are conducted at laboratory scale using small batch reactors, where microwave fields are relatively uniform and controllable. However, scaling up microwave systems introduces issues such as uneven electromagnetic field distribution, limited penetration depth, and inefficient energy coupling in large-volume materials. Future efforts should focus on the development of continuous-flow microwave reactors, optimized cavity designs, and hybrid heating systems that combine microwave irradiation with conventional heating. Advanced modeling and simulation of microwave-matter interactions will also play an essential role in designing industrial-scale microwave reactors with improved energy efficiency and process stability.

### 5.2 Mechanistic understanding of microwave-matter interactions

Although microwave heating has demonstrated significant advantages in accelerating chemical reactions, the underlying mechanisms governing microwave-matter interactions remain insufficiently understood. In particular, the existence and contribution of potential non-thermal microwave effects continue to be debated. A deeper understanding of how microwave irradiation influence reaction kinetics, diffusion processes, and phase transformations in complex battery materials is crucial for optimizing recycling processes. Future studies should combine advanced *in situ* characterization techniques, such as synchrotron X-ray diffraction, Raman spectroscopy, and thermal imaging, with theoretical modeling to elucidate the fundamental mechanisms of microwave-enhanced reactions.

### 5.3 Integration with green and sustainable recycling processes

Another promising direction is the integration of microwave technology with emerging green recycling strategies. For instance, microwave-assisted processes can be combined with environmentally benign leaching agents such as organic acids, DES, and bio-based solvents to reduce the environmental footprint of hydrometallurgical recycling. Furthermore, microwave-assisted carbothermal reduction and selective lithium extraction offer opportunities to simplify process flows and reduce reagent consumption. Developing closed-loop recycling processes that minimize chemical waste and maximize material recovery will be essential for achieving sustainable battery recycling systems.

### 5.4 High-value direct regeneration of electrode materials

Beyond conventional metal extraction routes, microwave technology shows great potential for the direct regeneration of electrode materials. Direct recycling approaches aim to restore the structure and electrochemical performance of degraded cathode and anode materials without completely breaking down their chemical composition. Microwave-assisted regeneration has already demonstrated promising results in repairing graphite anodes and restoring cathode materials such as LiCoO_2_ and LiFePO_4_. Future research should explore strategies for precise structural reconstruction, defect healing, and lithium replenishment in complex cathode materials, including high-nickel layered oxides and lithium-rich cathodes. These approaches could significantly reduce energy consumption while preserving the intrinsic value of battery materials.

### 5.5 Techno-economic and life-cycle assessment

Finally, comprehensive techno-economic analysis (TEA) and life-cycle assessment (LCA) are essential to evaluate the industrial feasibility of microwave-assisted recycling technologies. While laboratory studies indicate substantial reductions in energy consumption and reaction time, the economic viability and environmental benefits at large scale remain to be fully quantified. Future studies should systematically assess the cost, carbon footprint, and resource efficiency of microwave-assisted processes compared with conventional recycling routes. Such analyses will provide critical guidance for policymakers and industry stakeholders in developing sustainable battery recycling infrastructure.

Overall, microwave-assisted technologies offer substantial advantages in terms of reaction efficiency, energy consumption, and environmental performance, making them highly promising for the development of sustainable and high-efficiency LIB recycling processes. Although challenges related to reactor design and large-scale implementation remain, microwave heating provides a powerful strategy for overcoming the limitations of conventional recycling technologies and advancing the development of next-generation battery recycling systems.
